# The identification, adaptive evolutionary analyses and mRNA expression levels of homeobox (*hox*) genes in the Chinese mitten crab *Eriocheir sinensis*

**DOI:** 10.1186/s12864-023-09489-w

**Published:** 2023-08-03

**Authors:** Shasha Chen, Xianfeng Jiang, Longjie Xia, Zhiyi Chen, Kaiya Zhou, Jie Yan, Peng Li

**Affiliations:** Jiangsu Key Laboratory for Biodiversity and Biotechnology, College of Life Sciences, Nanjing Normal University, Nanjing, 210023 China

**Keywords:** *Eriocheir sinensis*, Homeobox genes, Developmental regulation, Adaptive evolution

## Abstract

**Background:**

Arthropods are the largest group in the animal kingdom and are morphologically characterized by heterorhythmic segments. Brachyuran decapod crustaceans undergo brachyurization metamorphosis in the early developmental process, characterized by a reduced abdomen that is folded beneath the cephalothorax and inserted between the pereiopods or in a special cavity. As the main cause of major alterations in the evolution of animal body plans, *Hox* genes encode transcription factors and are involved in bilaterian anterior-posterior axis patterning.

**Results:**

We found eight *Hox* genes (*labial*, *proboscipedia*, *Deformed*, *zerknüllt*, *Sex combs reduced*, *Antennapedia*, *Ultrabithorax*, *fushi tarazu*, *abdominal-A* and *Abdominal-B*) in *Eriocheir sinensis.* The phylogenetic topology of 13 arthropod *Hox* genes was closely related to traditional taxonomic groupings. Genome collinearity analysis was performed using genomic data and chromosomal location data of *E. sinensis* and *Portunus trituratus*. We found that their chromosomes were highly collinear, and there was a corresponding collinear relationship between the three *Hox* genes (*lab, ftz* and *Abd-B*). The mRNA expression levels of *Scr* and *Antp* fluctuated significantly in different developmental stages of *E. sinensis*, especially in the brachyurization stages. Evolutionary analysis indicated the presence of positively selected sites in *Ubx*.

**Conclusions:**

In this study, we used genome-wide analysis to identify and analyze all members of the *Hox* genes in *E. sinensis*. Our data will contribute to a better understanding of *Hox* genes in *E. sinensis* and provide useful molecular evolutionary information for further investigation on their roles in the brachyurization of crabs.

**Supplementary Information:**

The online version contains supplementary material available at 10.1186/s12864-023-09489-w.

## Highlights

Eight *Hox* genes (*lab*, *Dfd*, *Scr*, *ftz*, *Antp*, *Ubx*, *abd-A*, and *Abd-B*) were identified in *Eriocheir sinensis*.

There was a corresponding collinear relationship between the three *Hox* genes (*lab, ftz* and *Abd-B*) in *E. sinensis* and *Portunus trituratus*.

Positively selected sites in *Ubx* (1 M 0.952*, 3 S 0.998**, 4 Y 0.998**, P = 0.0000) were identified in *E. sinensis.*

The full-length cDNA of *Scr* and *Antp* in *E. sinensis* were cloned and characterized. *Hox* genes (*Scr* and *Antp*) were differentially expressed during the larval development of *E. sinensis*.

**Supplementary Information:**

The online version contains supplementary material available at 10.1186/s12864-023-09489-w.

## Introduction

In the lifecycle of an organism, the formation of the body and its organs during ontogeny or regeneration is known as morphogenesis. Homeobox genes are essential for controlling embryonic patterning and regulating these anatomical changes. These genes are mainly characterized by an approximately 180 bp (bp) homeodomain conserved region [1]. Homeobox genes were first identified in *Drosophila melanogaster* [2], followed by sequential discovery in all multicellular organisms, from parasites to vertebrates, plants and fungi, and they are highly evolutionarily conserved [3, 4].

Traditionally, homeobox genes are divided into two subfamilies. One contains genes that are arranged in clusters on chromosomes and expressed along the main anterior-posterior (A-P) axis of the animal body, which are called *Hox* genes or type-I homeobox genes. The other are non-A-P homeobox genes, which are not arranged in clusters but rather are scattered across different chromosomes, and they are named type-II homeobox genes based on sequence similarity [5]. In particular, *Hox* genes are specifically related to the regulation of body plan development during the embryonic stage of the life cycle [6]. *Hox* genes are mostly involved in the morphogenesis and differentiation of tissues or organs such as limbs [7], brain [8], muscle [9], blood [10], and bone [11]. Each *Hox* gene contains a conserved homeobox domain (homeodomain, HD) involved in the regulation of downstream genes [12]. The homeobox domain is a 60-amino-acid protein sequence with an alpha-helical secondary structure [13]. The activity of these regulatory proteins encoded by homeobox genes is most pronounced at the early embryonic stage, when the embryo is forming a body axis [14]. The expression patterns of *Hox* genes show a linear pattern: genes at the 3’-end are expressed first and regulate the anterior development of the embryo, while genes at the 5’-end are expressed later and regulate the posterior development of the embryo [15]. This phenomenon is called spatial collinearity and temporal collinearity.

Arthropods have maximum species richness in the animal world, and they are also the most morphologically diverse group, accounting for approximately 80% of all animal species [16]. To date, ten ancestral homologs have been confirmed in arthropods: *labial* (*lab*), *proboscipedia* (*pb*), *Deformed* (*Dfd*), *zerknüllt* (*zen*), *Sex combs reduced* (*Scr*), *Antennapedia* (*Antp*), *Ultrabithorax* (*Ubx*), *fushi tarazu* (*ftz*), *abdominal-A* (*abd-A*) and *Abdominal-B* (*Abd-B*) [17]. *Drosophila melanogaster* has eight *Hox genes* and was found to be divided into two homologous heterogeneous gene clusters on chromosome 3: the antennal foot complex Antp-C (antennapedia complex) gene cluster and the double-thorax complex BX-C (bithorax complex) gene cluster [18]. Antp-C is mainly involved in the developmental regulation of the head and chest, and BX-C is mainly involved in the regulation of posterior thoracic and abdominal development [19].

In the early developmental process, brachyuran decapod crustaceans undergo brachyurization metamorphosis, which is characterized by a reduced abdomen that is folded beneath the cephalothorax and inserted between the pereiopods or in a special cavity [20, 21]. The Chinese mitten crab (*Eriocheir sinensis*) is a limnic and intertidal crab mainly distributed in eastern and northern China. The juvenile development period of *E. sinensis* normally consists of five zoeae stages and a megalopa stage, and it subsequently enters the first juvenile crab stage and develops into an adult crab after multiple molts [22]. The most conspicuous feature of morphological change from megalopa to adult crab is brachyurization, including abdominal degeneration. A previous study showed that *Hox* genes are involved in embryonic development and appendage development in crustaceans [23], but the molecular regulatory mechanisms of brachyurization are poorly studied [24].

In this study, we used genome-wide analysis to identify all members of the *Hox* gene family between *E. sinensis* and 12 other arthropod species. These sequences were aligned and used to construct a phylogenetic tree. Moreover, the full-length cDNA of the *Scr* and *Antp* genes were cloned and characterized from the Chinese mitten crab (*E. sinensis)* using expressed sequence tag (EST) and rapid amplification of cDNA end (RACE) techniques. The mRNA expression profiles of *Scr* and *Antp* transcripts in various samples (different developmental stages of juvenile crabs) were measured by fluorescent real-time quantitative PCR (RT‒qPCR). The selective pressure of *Scr* and *Antp* was determined to reveal the relationship between brachyurization and adaptive evolution of *Hox* genes in Brachyura crabs. Our data will contribute to a better understanding of *Hox* genes in *E. sinensis* and provide useful molecular evolutionary information for further investigation on their roles in brachyurization of crabs.

## Result

### Identification and analyses of *Hox genes* in the Chinese mitten crab

Eight *Hox* genes (*lab*, *Dfd*, *Scr*, *ftz*, *Antp*, *Ubx*, *abd-A* and *Abd-B*) were identified through BLAST analysis of the *E. sinensis* genome database. A sequence alignment of the eight Hox proteins in *E. sinensis* with those of other species, such as *D. melanogaster* and *L. vannamei*, revealed the presence of a residue sequence of YPWM and a conserved domain of homeodomain except *Abd-B* (Supplementary file S1). We analyzed the physical properties and amino acid composition of the Hox proteins in Chinese mitten crab and found that it was rich in Ala, Gly, Pro, Gln, Ser and Thr (Supplementary file S2 and Supplementary file S3).

**Fig. 1:**
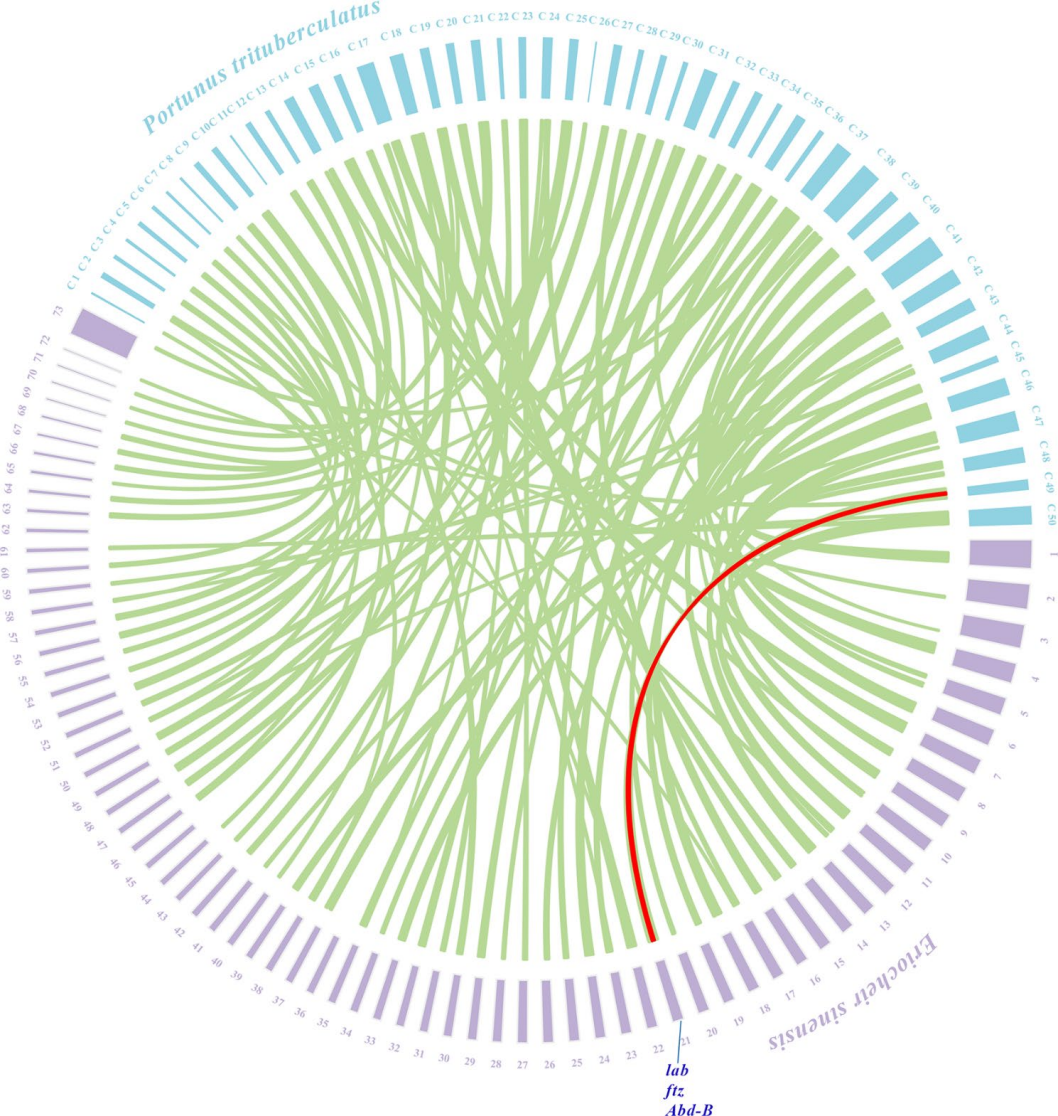
Collinearity patterns between genomic regions from *Eriocheir sinensis* and *Portunus trituratus*

The amino acid sequences of eight Hox proteins from *E. sinensis* were clustered. The results showed that the eight *Hox* genes were classified into two major clades. *Scr*, *Dfd*, *ftz*, *Ubx* and *abd-A* clustered together, and the other group included *Antp*, *Abd-B* and *lab*. The genomes of *E. sinensis* and *P. trituratus* were analyzed for collinearity, and there was a high degree of collinearity among multiple chromosomes **(**Fig. 1**)**. This reflects the sister group relationship of *E. sinensis* and *P. trituratus*. Among them, the *Hox* genes *(lab, ftz* and *Abd-B*) present on chromosome 21 in *E. sinensis* are colinear with those in the *P. trituratus* genome, retaining more ancestral traits.

### Phylogenetic analysis of the *Hox* gene family

**Fig. 2:**
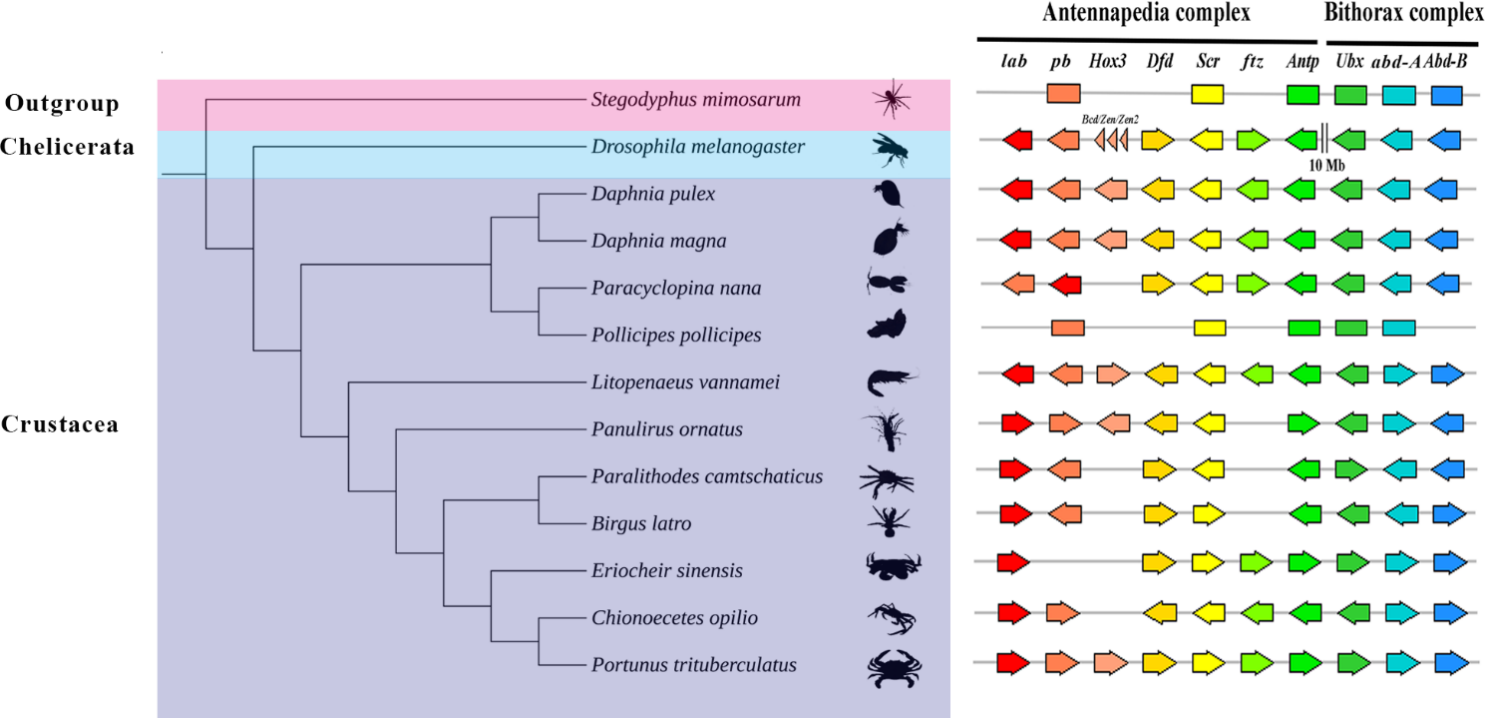
Comparison of Hox genes among arthropods

**Fig. 3:**
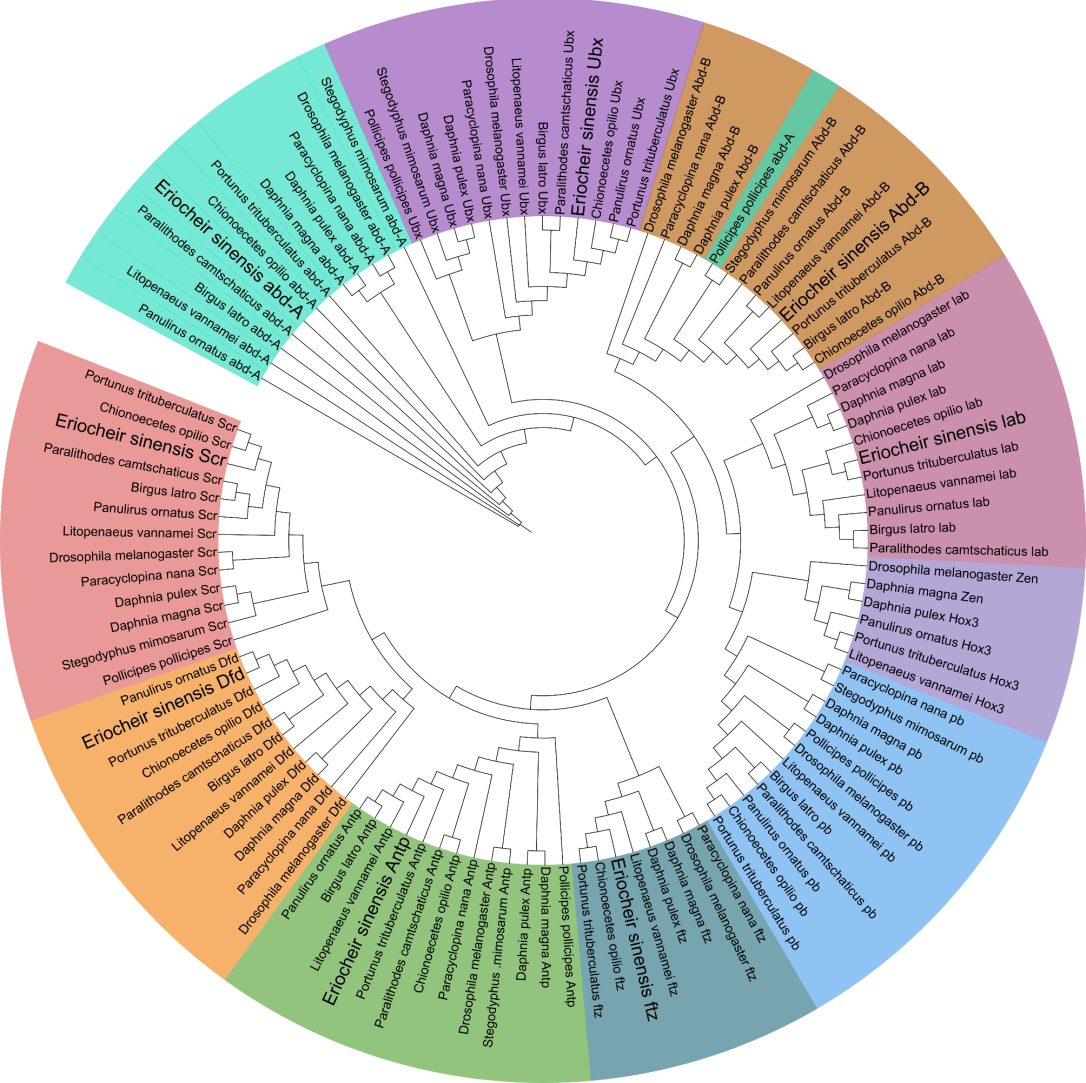
Phylogenetic tree of Hox among arthropods

We found a total of 112 *Hox* genes in 13 selected species and mapped their transcription orientation from genomic data with annotation files (Fig. 2). Aligning Hox protein sequences, a total of 112 Hox protein sequences from 13 selected species were used to build a phylogenetic tree using the maximum likelihood approach (Fig. 3, Supplementary file S5). *E. sinensis* and *P. trituberculatus*, belonging to Eubrachyura, are sister groups to shrimps and grouped with hermit crabs into one class. Each *Hox* gene of *Eriocheir sinensis* was first clustered with the *Hox* gene of crabs and shrimps. This clustering phenomenon shows that crustaceans are relatively closely related to insects and slightly distantly related to Chelicerata species. The results support the traditional taxonomy.

### Adaptive evolution analyses of *Hox* genes

Different ω ratio models were used to determine if *Hox* genes in the arthropod species have experienced positive selection. In the site model, the positively selected site with a posterior probability greater than 0.95 was identified only in *Ubx.* In the branching model, *E. sinensis* was set as the foreground branch, and the other branches were set as background branches. Out of the seven *Hox* genes (*lab, Scr, Antp, Ubx, ftz, abd-A* and *Abd-B*), the results showed that there was a significant difference in the evolution rate of *Ubx* and *abd-A* between *E. sinensis* and other animals, so *Ubx* and *abd-A* were considered rapidly evolving genes. The results of branch-site model A revealed evidence of positive selection on *Ubx* (1 M 0.952*, 3 S 0.998**, 4 Y 0.998**) among the arthropod lineages (Tables 1, 2 and 3). Setting Brachyura as the foreground branch for selection pressure analysis, we found that in the branch model, the evolution rate of *Ubx* was different in Brachyura from the background branch. In the branch site model, *Antp* (57 K 1.000**) had a positive selection site, and its mutation site was the same as the positive selection site of the Chinese mitten crab. This indicates that the *Antp* gene is possibly involved in the brachyurization development of crabs (Supplementary file S6 and Supplementary file S7).

**Table 1:** Selective pressure analysis of *Hox* based on site model

Gene	Models	np	-lnL	LRT P- values	positively selected sites (*PP* > 0.95)
*lab*	M7 M8	23 25	1453.233 1451.769	0.2312	Not allowed ————
*Scr*	M7 M8	26 28	3338.035 3318.876	0.0000	Not allowed 98 Q 0.802
*Antp*	M7 M8	24 26	1394.409 1394.409	0.9993	Not allowed ————
*Ubx*	M7 M8	18 20	2034.683 2030.628	0.0173	Not allowed 120 T 0.999*
*ftz*	M7 M8	16 18	4599.532 4599.532	0.9998	Not allowed 57 P 0.533
*abd-A*	M7 M8	18 20	2365.844 2365.845	0.9990	Not allowed ————
*Abd-B*	M7 M8	21 23	2656.995 2656.995	0.9997	Not allowed ————

**Table 2:** Selective pressure analysis of *Hox* based on branch model

Gene	Models (branch *E. sinensis*)	np	-lnL	LRT P- values	Omega values
*lab*	one-ratio (M0) two-ratio	22 23	1455.098 1454.546	0.2931	ω_0_ = 0.0850 ω_1_ = 0.0001
*Scr*	one-ratio (M0) two-ratio	25 26	3344.156 3343.366	0.2087	ω_0_ = 0.1283 ω_1_ = 0.6928
*Antp*	one-ratio (M0) two-ratio	23 24	1464.302 1463.961	0.4098	ω_0_ = 0.0509 ω_1_ = 0.4207
*Ubx*	one-ratio (M0) two-ratio	17 18	2067.615 2062.555	0.0015	ω_0_ = 0.0336 ω_1_ = 0.2125
*ftz*	one-ratio (M0) two-ratio	15 16	4713.532 4713.434	0.6572	ω_0_ = 0.1134 ω_1_ = 0.1601
*abd-A*	one-ratio (M0) two-ratio	17 18	2359.625 2359.425	0.5271	ω_0_ = 0.0047 ω_1_ = 0.0001
*Abd-B*	one-ratio (M0) two-ratio	20 21	2680.479 2681.455	0.1622	ω_0_ = 0.0232 ω_1_ = 20.9758

**Table 3:** Selective pressure analysis of *Hox* based on branch site model

Gene	Models (branch *E. sinensis*)	np	-lnL	LRT P- values	positively selected sites (*PP* > 0.95)
*lab*	Model A Model A null	25 24	1452.449 1452.449	1.0000	———— Not allowed
*Scr*	Model A Model A null	28 27	3316.316 3316.316	1.0000	21 G 0.943,45 S 0.937
*Antp*	Model A Model A null	26 25	1392.478 1394.476	0.0456	56 M 0.805,57 K 0.961*
*Ubx*	Model A Model A null	20 19	2037.891 2045.864	0.0001	1 M 0.960*, 3 S 0.998**, 4 Y 0.998**, 5 E 0.552, 6 Q 0.684
*ftz*	Model A Model A null	18 17	4640.752 4640.752	0.9989	———— Not allowed
*abd-A*	Model A Model A null	20 19	2359.625 2359.625	0.9989	———— Not allowed
*Abd-B*	Model A Model A null	23 22	2678.448 2678.448	0.9975	———— Not allowed

### Full-length cDNA structure of *Scr* and *Antp* and mRNA expression of the *Antp* and *Scr* genes in *E. sinensis* during different developmental periods

The full-length cDNA sequences of *Scr* (1770 bp, **KP822930**) and *Antp* (2413 bp, **KP822927**) were obtained by DNAStar Lasergene 7.1 after the sequence splicing of the *Scr* and *Antp* genes. Through analysis, it was found that the full-length cDNA sequence of *Scr* has a 5’-end untranslated region (UTR) of 424 bp and a 3’-end untranslated region of 350 bp, and its open reading frame (ORF) contains 996 bp, encoding 332 amino acids. The full-length cDNA sequence of *Antp* has a 5’-end untranslated region (UTR) of 324 bp and a 3’-end untranslated region of 1123 bp, and its ORF contains 966 bp, encoding 322 amino acids. Their open reading frames both contain a conserved domain (Homeobox) (Supplementary files S4).

**Fig. 4:**
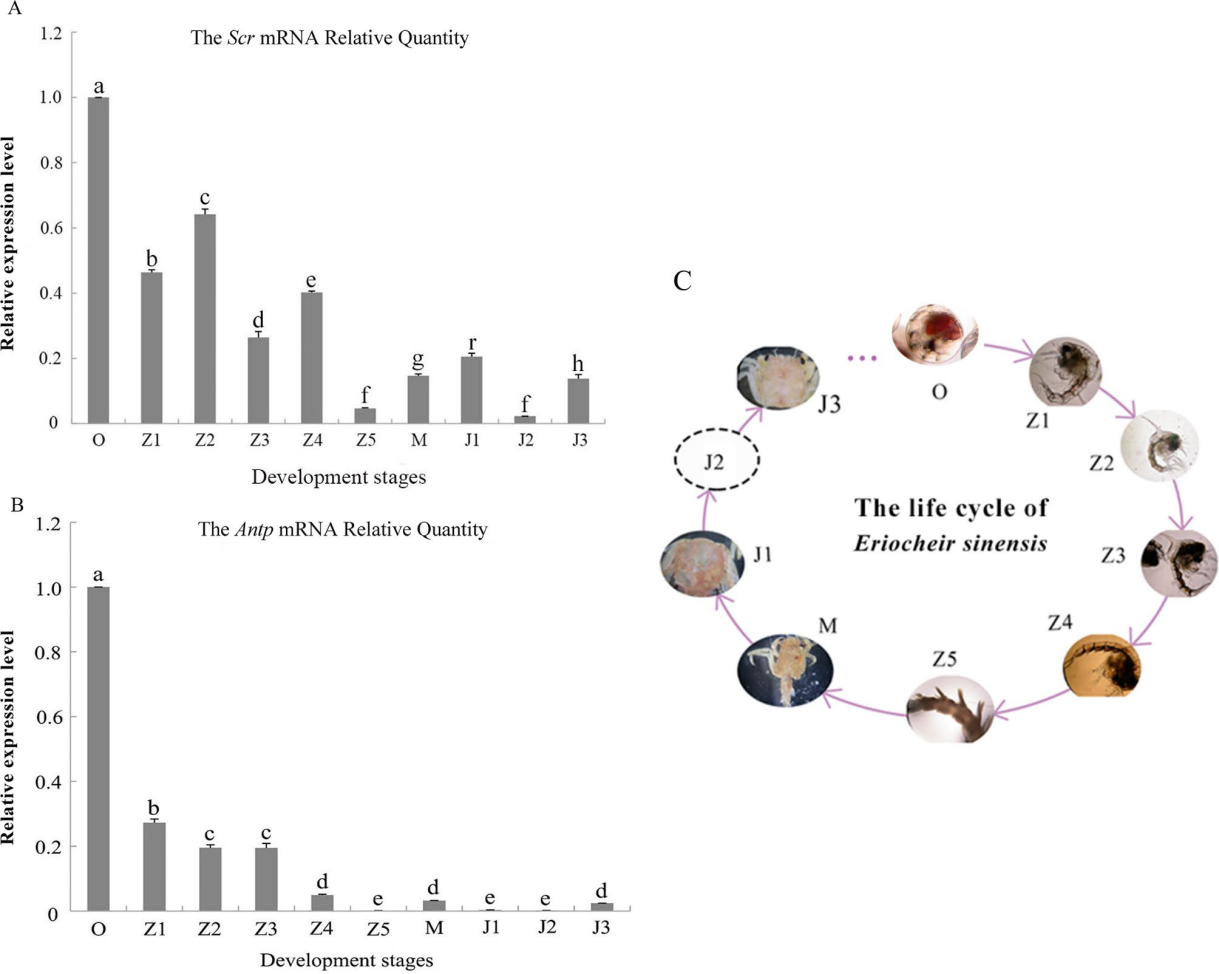
The developmental periods of *Eriocheir sinensis*. A, B : the relative mRNA expression of *Antp* and *Scr* of *Eriocheir sinensis* in different development stages (n = 9, mean ± SD). C: the pictures of life cycle of the Chinese mitten crab photoed by Peng Li

The mRNA transcripts of *Scr* and *Antp* were detected in all tested developmental stages of the Chinese mitten crab. The mRNA expression of *Scr* in *E. sinensis* was relatively higher at stages O and Z1 to Z4 and lower at stages Z5, M and J1 to J3 (Fig. 4A**)**. The mRNA expression level of *Antp* in *E. sinensis* was the highest at stage O, and the expression of *Antp* gradually decreased with the development and growth of crabs (Fig. 4B**)**.

## Discussion

In this study, we identified a single *Hox* gene cluster including seven typical homeotic genes and one additional homeotic gene from the genome of *E. sinensis*. However, orthologs of *pb* and *Hox3* were not found in *E. sinensis* based on comparison with the sequences of arthropods, including *D. melanogaster* and *L. vannamei*. However, because 10 *Hox* genes were extracted from the transcriptome of the Chinese mitten crab in a previous study [25], the *pb* and *Hox3* genes were not found in this study, perhaps due to incomplete genomic assembly for *E. sinensis*.

Previous studies have found that the repertoire of *Hox* genes differs among different species. *D. melanogaster*, *L. vannamei*, *Daphnia pulex* and *Daphnia magna* are represented by complete sequences for all ten *Hox* genes [26, 27]. However, only nine *Hox* genes from *Paracyclopina nana* were identified, all except *Hox3* [28]. A *Hox3* gene ortholog was also not present in the *Hox* gene cluster of *Macrobrachium olfersii* [29]. Moreover, *pb*, *Hox3* and *ftz* were missing in two species of copepods, *Tigriopus japonicus* and *T. kingsejongensis* [30]. Because *Hox* gene clusters are expressed along the main anterior-posterior (A-P) axis, dynamic mutation in the *Hox* gene causes the ectopic development of a given organ. Normal development of *D. melanogaster* results in a pair of balancing rods in the posterior thorax, but a mutation of *Ubx* will cause flies to grow a pair of wings in the posterior thorax [31]. The data of *Hox* gene sequences in arthropods, especially crustaceans, will contribute to explaining the common morphological changes and evolutionary process in crustaceans during metamorphosis.

Compared with higher vertebrates, studies on *Hox* genes in arthropods lag far behind. *Hox* genes have been reported to play important roles in cell division, paired appendage development, axial morphogenesis regulation and skeleton maintenance [32, 33]. The basal expression levels of *Hox* genes demonstrated different patterns of expression over different developmental stages. We selected *Scr* and *Antp* for further investigation because we only cloned these two *Hox* genes (failed to acquire the full-length cDNA of other *Hox* genes in *E. sinensis*) in the early stage of our experiment (before the genome of *E. sinensis* was determined) and examined the mRNA expression patterns of these two genes. In this experiment, the mRNA expression patterns of the *Scr* and *Antp* genes were detected at all 10 stages of juvenile development in *E. sinensis*, suggesting that *Scr* and *Antp* may play a regulatory role in the juvenile development of *E. sinensis*. Previous studies indicated that *Scr* controls the development of the insect prothorax [34], and *Antp* is involved in regulating morphological changes in the abdominal appendages in crustaceans [35, 36]. The main morphological changes in brachyurization development mainly occurred in stages Z5, M and J1 of *E. sinensis* (Fig. 4C), and *Scr* and *Antp* mRNA were expressed at the lowest levels in stages Z5 ~ J3, indicating the possibility that these *Hox* genes are partially involved in brachyurization and further developmental regulation. The thoracic function of *E. sinensis* is increased with shortened abdomen, whereas the abdominal function is weakened, certain abdominal segments heal and fold under the cephalothorax, and the motor function is lost. The mRNA expression of *Scr* and *Antp* changed dramatically during their respective larval developmental phases, mirroring changes in the morphology of *E. sinensis* larvae. During the egg stage, when the Chinese mitten crab individuals were fully formed, the relative expression of the *Scr* and *Antp* gene mRNAs peaked. In contrast, during the Z5 stage, their expression reached its lowest point, which caused abdominal degeneration in *E. sinensis*. This significant change (*p* < 0.01) implied that these two genes might be involved in regulating the short tailing process of *E. sinensis*. Young et al. indicated that *Hox* genes are highly expressed in the vertebrate posterior embryonic region and play a role in posterior tissue generation [37]. This finding fits with the so-called dose effect that quantitative variation in the levels of *Hox* gene products can affect segment morphology in subtle ways [38].

The rate of gene evolution depends on the substitution rate of the nucleotides in the nucleic acid molecule over a certain period of time, and the selection pressure reacts to the rate of gene evolution. Based on selective pressure analyses of *Hox* genes, *E. sinensis* was set as the foreground branching clade in the branching model and branch site model. The results revealed that six *Hox* genes had no positive selection signal, but *Antp and Ubx* did, indicating that the evolutionary rates of *Antp and Ubx* were perhaps different among different species. A previous study reported that in the early course of gene evolution, the evolutionary rate of the *Hox* gene in actinopterygian and sarcopterygian fishes is faster than that in terrestrial vertebrates [39]. The different rates of *Hox* gene evolution imply that *Hox* genes may regulate animal morphology to adapt to the environment. Overall, there have been a relatively detailed investigations of the evolution and expression pattern of *Hox* genes in Brachyura crabs, but further work is needed to focus on the molecular evolutionary mechanism and functions in developmental regulation of brachyurization.

## Conclusion

Here, we explored eight *Hox* genes from the chromosomal level genome of the Chinese mitten crab *E. sinensis.* A total of 112 putative *Hox* genes were identified in 13 arthropod species. According to the collinear analysis, there are a large number of collinear fragments between *E. sinensis* and *P. trituratus*, which implies that they accumulate less variation with shorter differentiation times and retain more ancestral traits. Evolutionary analyses indicated the presence of positively selected sites in *Ubx*. In the branch model, the *Ubx* and *abd-A* genes in *E. sinensis* were identified as rapidly evolving genes. The full-length cDNA sequences of *Scr* and *Antp* were cloned, and the mRNA expression patterns of the two *Hox* genes in *E. sinensis* were determined at different developmental stages. The expression levels of *Scr* and *Antp* in *E. sinensis* were the highest at stage O and fluctuated significantly in different developmental periods. Our data will contribute to a better understanding of the *Hox* gene family in *E. sinensis* and provide useful molecular evolutionary information for further investigation into their roles in the brachyurization of crabs.

## Materials and methods

### Sample collection

Tissue samples of *E. sinensis* were collected from the healthy Chinese mitten crabs that were bred in an aquatic nursery of Rudong County, Nantong City, Jiangsu Province, China. The samples were collected at the fertilized egg stage (stage O), all zoea stages (stage Z1 ~ Z5), megalopa stage (stage M) and juvenile crab stages (stage J1 ~ J3, with approximately 30 mg for each sample). These fresh tissues were stabilized immediately in RNA*later* RNA Stabilization Reagent (QIAGEN, Germany), kept overnight at + 4 °C, and finally stored at − 20 °C before RNA isolation.

### Preparation of total RNA

Following the manufacturer’s instructions, we used an RNeasy Mini Kit (Qiagen, Germany) to extract total RNA from different samples from the Chinese mitten crab. The RNA concentration and purity were assessed spectrophotometrically by measuring the absorbance of the solution at 260 and 280 nm in a biophotometer (Eppendorf, Germany). The RNA fragmentation state was evaluated by 1% agarose denatured gel electrophoresis.

### Phylogenetic analysis of *Hox* genes

For comparative genomics analyses, we used all the annotated genes in the Chinese mitten crab and thirteen other arthropod species. Genome assemblies of 13 species were obtained from the National Center for Biotechnology Information (NCBI, https://www.ncbi.nlm.nih.gov/) and GigaDB database (http://gigadb.org/) (Table 4). The Hox amino acid sequences of *Litopenaeus vannamei* and *Drosophila melanogaster* were used as queries. BLASTP in BLAST v2.2.23 was used for similarity searches in the genomes of the selected species. An e-value of 10^− 5^ was used as an initial cutoff to identify significant matches. Hits < 100 codons and overlapping sequences were excluded.

**Table 4:** The GenBank accession used in this study

subphylum	phylum	scientific name	accession
Crustacea	Malacostraca	*Eriocheir sinensis*	GCA_003336515.1
	Malacostraca	*Chionoecetes opilio*	GCA_016584305.1
	Malacostraca	*Portunus trituberculatus*	GCA_008373055.1
	Malacostraca	*Birgus latro*	GCA_018397915.1
	Malacostraca	*Paralithodes camtschaticus*	GCA_018397895.1
	Malacostraca	*Litopenaeus vannamei*	GCA_003789085.1
	Malacostraca	*Panulirus ornatus*	GCA_018397875.1
	Branchiopoda	*Daphnia magna*	GCA_020631705.2
	Branchiopoda	*Daphnia pulex*	GCA_021134715.1
	Copepoda	*Paracyclopina nana*	GCA_019096065.1
	Maxillopoda	*Pollicipes pollicipes*	GCA_011947565.3
Chelicerata	Arachnida	*Stegodyphus mimosarum*	GCA_000611955.2
Hexapoda	Insecta	*Drosophila melanogaster*	GCA_003401745.1

The ProtParam software tool at the ExPASy portal (https://web.expasy.org/protparam/) was used to calculate the amino acid percentages of Hox gene-encoded proteins. CLUSTALW 2.0.10 [40] was used to compare the homology of Hox protein sequences in *E. sinensis*, *D. melanogaster* and *L. vannamei*. The gene structure of the *Hox* genes and the distribution of motifs and domains at each sequence were visualized using TBtools version 1.074 [41]. Mapping of gene family members in chromosome locations was performed using the R 4.0.5 package (“ggplot2” and “gggenes”) [42]. Amino acid sequences were aligned with MAFFT, and phylogenetic analyses were performed with IQtree on the Phylosuit platform. The phylogenetic analysis of Hox protein sequences used the JTT model for maximum likelihood analysis with 1000 bootstrap replicates. Using genomic data and chromosomal location data of *E. sinensis* and *Portunus trituratus*, cross-species genome collinearity analysis was performed using MCScanX software and then visualized in Circos 0.67.

### Analyses of evolutionary pressure

We used comparisons of nonsynonymous/synonymous substitution ratios (dN/dS) to quantify natural selection. Different types of selection were represented by different values: ω < 1 means purifying selection, ω = 1 indicates neutral selection, and ω > 1 means positive selection. Due to the incomplete sequence of *Dfd*, we only performed selective pressure analysis on the other seven *Hox* genes (*lab, Scr, Antp, Ubx, ftz, abd-A* and *Abd-B*) in *E. sinensis*. Analyses of selective pressure were conducted using the Codeml program implemented in the Phylogenetic Analysis by Maximum Likelihood (PAML4.9) package. Different *Hox* genes in crustacean species have experienced positive selection. The selected site model (Site model), branch model (Branch model), and branch site model (Branch-site model) were used to test for the presence of positive branches on the divergent clades of the crustaceans for *Hox*.

### Full-length cDNA amplification and the mRNA expression patterns of *Antp* and *Scr* during different periods

Gene-specific primers for *Scr* and *Antp* gene amplification were designed based on the high-throughput sequencing of the Chinese mitten crab transcriptome sequence obtained earlier in our laboratory (data unpublished). The primers are shown in Table 5. The cDNA was synthesized using a 3’ Full RACE Core Set with PrimeScript™ RTase (Qiagen, Germany). PCR amplification was followed by using TaKaRa LA *Taq* with GC Buffer (TaKaRa, Dalian, China). The *β-actin* gene (GenBank accession no. **HM053699.1**) of *E. sinensis*, as an effective internal control [26, 43], was selected to calibrate the cDNA template. Gene-specific primers for RT‒qPCR were designed according to the sequences of the two genes (*Scr* and *Antp*), and these primers are shown in Table 5. Real-time quantitative PCR (RT‒qPCR) was conducted using the SYBR^®^ Premix *Ex*-Taq™ II kit (TaKaRa, Dalian, China). The RT‒qPCR cycling conditions were as follows: initial denaturation at 95 °C/30 sec; 40 cycles of 95 °C/5 sec and 60 °C/30 sec; 95 °C/15 sec; 60 °C/1 min, 95 °C/15 sec. The RT‒qPCR assay was carried out in triplicate on 96-well plates. Employing the formula RATE = 2^−ΔΔCt^, the comparative Ct method was used to analyze the relative expression levels of *Scr* and *Antp*. Statistical analysis of the *Scr* and *Antp* gene expression data was performed using STATISTICA 10.0. One-way analysis of variance (ANOVA) with Tukey’s post hoc tests were performed to assess statistical significance, in which statistical significance was accepted at *p* ≤ 0.05 and marked with asterisks or different letters.

**Table 5:** Primers used for *Scr* and *Antp* genes amplification and real-time quantitative PCR detection

primer name	primer sequences (5’→3’)	primer length (bp)
Scr-F0	5’-CAGATCTACCCGTGGATGAAGAG-3’	23
Scr-R0	5’-CGTTCATGCTCGCCATCTTGTGC-3’	23
Scr-F1	5’-GAACTCCAACGGCGAGACCAAG-3’	22
Scr-R1	5’-CCATCTTGTGCTCCTTCTTCCA-3’	22
Scr-F2	5’-CCTATTGGTTGCTGTCGGTCAC-3’	22
Scr-R2	5’-ATATTTTGATCTGTCGCTCGGT-3’	22
Scr-R3	5’-GCGTAGTCCTGAAGGGTAGTCCAT-3’	24
Antp-F0	5’-CAGCAACAGCAGGCACAGCAACA-3’	23
Antp-R0	5’-CTGCGAGGGGGACGTTGGGGTC-3’	22
Antp-F1	5’-CCAGGATACCTCCCTCCACATG-3’	22
Antp-R1	5’-GGGTCGGCGTATCTGAAAGGCT-3’	22
Antp-R2	5’-TGCTCTTGTTTTCTTTCTTCCA-3’	22
Antp-R3	5’-AGGGCCGATGAGGTGTTTTGTT-3’	22
Antp-F2	5’-TCATAGTGTAAAGTGTATTCTGT-3’	23
Antp-F3	5’-CGGGAAACAGCCTACCAGTGCC-3’	22
Antp-F4	5’-CACGCCGACTCCTGTCATCCCT-3’	22
Antp-F5	5’-CAAGAGCAAAGTGGAGAACGGGAACA-3’	26
Antp-R4	5’-CAAACAATACCAGAGTAGGAGAAGC-3’	25
Antp-R5	5’-GTCTCGTCCGCTTCTCGTTCCTG-3’	23
primer name	primer sequences (5’→3’)	primer length (bp)
QEs-ScrF	5’-GAGTCTGGAAGGAGCCTCTG-3’	20
QEs-ScrR	5’-CGGGTAGATCTGTGGTTGTG-3’	20
Qβ-actinF	5’-CTCCTGCTTGCTGATCCACATC-3’	22
Qβ-actinR	5’-GCATCCACGAGACCACTTACA-3’	21
QEs-AntpF	5’-ATTCCACTTCAACCGCTACC-3’	20
QEs-AntpR	5’-CTGTTCCCGTTCTCCACTTT-3’	20
Qβ-actinF	5’-CTCCTGCTTGCTGATCCACATC-3’	22
Qβ-actinR	5’-GCATCCACGAGACCACTTACA-3’	21

### Electronic supplementary material

Below is the link to the electronic supplementary material.

Supplementary Material 1

Supplementary Material 2

Supplementary Material 3

Supplementary Material 4

Supplementary Material 5

Supplementary Material 6

Supplementary Material 7

## Data Availability

All data generated or analysed during this study are included in this published article.
