# Supplementary material for: The identification, adaptive evolutionary analyses and mRNA expression levels of homeobox (hox) genes in the Chinese mitten crab Eriocheir sinensis
Source: BMC Genomics. 2023 Aug 3;24:436. doi: 10.1186/s12864-023-09489-w (PMC10401747; doi:10.1186/s12864-023-09489-w)
Supplement: Supplementary file 1 — Supplementary Material 1 [file 12864_2023_9489_MOESM1_ESM.pdf]

SUPPLEMENTARY FILE S1. Multiple sequences alignment of Hox proteins in Chinese mitten crab and other arthropods species.

```
                                                         1        10        20
E.sinensis_lab        ...................................MNTTNMVYGVCNAESGYHSQPYCSE
L.vannamei_lab        ...................................MNNTNMVYGVCNAESGYHSQAYSSE
D.melanogaster_lab    ............................................................
E.sinensis_Dfd        ............................................................
L.vannamei_Dfd        ............................................................
D.melanogaster_Dfd    ............................MSSFLMGYPHAPHHVQSPMSMGNGLDPKFPPL
E.sinensis_Scr        ..........................................MSSYQFVNSMSACYGQQR
L.vannamei_Scr        ...........................................MSSYQFVNSMSACYGQR
D.melanogaster_Scr    MDPDCFAMSSYQFVNSLASCYPQQMNPQQNHPGAEIRVQAARVEGRAVRGAWCQRRNERR
E.sinensis_ftz        ......................................................MSSYFA
L.vannamei_ftz        .................................................MSSYFANVPSP
D.melanogaster_ftz    .................................MATTNSQSHYSYADNMNMYNMYHPHSL
E.sinensis_Ubx        ....................................MSGSSMMDNFRALLQLAHAVHSPS
L.vannamei_Ubx        ............................................................
D.melanogaster_Ubx    ........................MNSYFEQASGFYGHPHQATGMAMGSGGHHDQTASAA
E.sinensis_Antp       ............................MFEGLMTVGCELAGGLPDAAPQHSTVAANSAP
L.vannamei_Antp       ............................................................
D.melanogaster_Antp   ....................MTMSTNNCESMTSYFTNSYMGADMHHGHYPGNGVTDLDAQ
E.sinensis_abd-A      ............................................................
L.vannamei_abd-A      ............................................................
D.melanogaster_abd-A  ............................................................
E.sinensis_Abd-B      ..........MNGSLYEDTASSRGLEPAGQTPLHIPAKRVATTLAVSTYPHPPECPTGPD
L.vannamei_Abd-B      ..........MNGALYEDSAGTRGLEPTGQTPLHIPAKRVATTLAVSTYPHPPECPTGPD
D.melanogaster_Abd-B  ............................................................

                         30        40        50
E.sinensis_lab        LQHYYGQCQSHEAVQGQTYIPPALTPTDHHR.............................
L.vannamei_lab        LQHYYAQCQSHEVVQGQTYIPPALTPTDHHR.............................
D.melanogaster_lab    ............................................................
E.sinensis_Dfd        ......MTMSSFLMNSGPYVDPKFPPPEEYS.............................
L.vannamei_Dfd        ......MTMSSFLMNSGPYVDPKFPPPEEYS.............................
D.melanogaster_Dfd    ADDYHHYNGHYSMTASTGHMSGAVGGGAGVGSVGGGGAGGMTGHPH..............
E.sinensis_Scr        GQDGSVVNTDYYGTSLNSYNNCYSPPLQYGG.............................
L.vannamei_Scr        GQDASGVNTDYYGASVNSYNNCYSPPLQQYG.............................
D.melanogaster_Scr    SGQCGGHSGGERLLPSRGAYTPNLYPNTPQAHYANQAAYGGQGNPDMVDYTQLQPLRLLL
E.sinensis_ftz        NVAAAGGWGAATTQEQWAYMQGSRGPAGYADVTQAYAQYGSGMQTY..............
L.vannamei_ftz        AANWGAPSQDQYAYMQSAKYPCYSDPAQAYA...QYGGMQGYRYPS..............
D.melanogaster_ftz    PPTYYDNSGSNAYYQNTSNYQGYYPQESYSESCYYY........................
E.sinensis_Ubx        APTRALRSLELNSTDPGGFYGGGSGGDQAYR.....FPLGLSVSPY..............
L.vannamei_Ubx        .........MNSYFEQGGFYGGGSGGDQAYR.....FPLGLSVSPY..............
D.melanogaster_Ubx    AAAYRGFPLSLGMSPYANHHLQRTTQDSPYDASITAACNKIYGDGA..............
E.sinensis_Antp       LAAYPPAPDMSHMSSYYNAYPDYRPPHPPDEYPGAPGGQGGGGAGG..............
L.vannamei_Antp       ...........MPPTHPYYTQQGYPRYPPYD.............................
D.melanogaster_Antp   QMHHYSQNANHQGNMPYPRFPPYDRMPYYNGQGMDQQQQHQVYSRP..............
E.sinensis_abd-A      ..............MSSNYIDSILPKYQAES.............................
L.vannamei_abd-A      ..............MSSNYIDSILPKYQADS.............................
D.melanogaster_abd-A  ...............MYPYVSNHPSSHGGLS.............................
E.sinensis_Abd-B      SGAAAGVIRHSHSTQPWNYQPDHAAATAPFDSQYGQAFYRDGVTPT..............
L.vannamei_Abd-B      SGTAAGVIRHSHSTQPWNYQPEHAAATAPFDSQYGQAFGRDGVTPT..............
D.melanogaster_Abd-B  ...........MSIQLAPLHIPAIRAGPGFE.............................

                         60        70        80
E.sinensis_lab        ..PQHTSLQDAHVPSSYTNLDDYSSVCYPGHA..........................AG
L.vannamei_lab        ..PQHTNLQDAHVPSSYTNLDDYSSVGYPGHA..........................AG
D.melanogaster_lab    ............................................................
E.sinensis_Dfd        ........QNSYIPPQSDYYNPAQHYSYHG...........MHQAG............MQ
L.vannamei_Dfd        ........QNSYIPPQSDYYNAAQHYPYHG...........MHQAPG...........MQ
D.melanogaster_Dfd    ..SMHPADMVSDYMAHHHNPHSHSHSHTHS...........LPHHHSNSAIS......GH
E.sinensis_Scr        ...YTP..SGISVVPNGSEFSAGGSSSASG...........TSASTASASS.......VG
L.vannamei_Scr        ..GYTP..SGLSAVPNGSEFPGGGSAGVVV...........TSASAGGSS........VG
D.melanogaster_Scr    QQQQQQQQQQLALAAAAVRAQQQQQLAQQQHPQQQQQQQQANISCKYANDPVTPGGSGGG
E.sinensis_ftz        ..RYPGAYAALNNAPATTKAAYEHSPTNNS...........PSPATTTSYDT......CS
L.vannamei_ftz        ..AYSC..ALGNMAATKGAYEQSPSPAITA............................TA
D.melanogaster_ftz    ..NNQEQVTTQTVPPVQPTTPPPKATKRKAEDDAASIIAAVEERPSTLRA........LL
E.sinensis_Ubx        ..GQPA........PRQDGYDASAAS................................CK
L.vannamei_Ubx        ..GQPG........PRQDGYDTTAAS................................CK
D.melanogaster_Ubx    ..GAYK..QDCLNIKADAVNGYKDIWNTGG...........SNGGGGGGGGGG.....GG
E.sinensis_Antp       ..GHCGQEYDPRMPPTHPYYTQQGYPRYPP.......YDRLMNNYYNAQTPQHPHTPHGM
L.vannamei_Antp       ...........RLMNNYYNAQTPQHPQTPH............................GM
D.melanogaster_Antp   ..DSPSSQVGGVMPQAQTNGQLGVPQQQQQQQQQPSQNQQQQQAQQAPQQLQQQLPQVTQ
E.sinensis_abd-A      ............AAANLVNYNTQARSMYP.............................YV
L.vannamei_abd-A      .............AANLVNYNSQARSMYP.............................YV
D.melanogaster_abd-A  .................GMAGFTGLEDKS.............................CS
E.sinensis_Abd-B      ..YYNI..ADSRMADRKALAFWPNKCEYAP...........GPSSMATES........CQ
L.vannamei_Abd-B      ..YYNI..ADTRVADRKTLAFWPNKYDYAP...........GPSSMTTES........CQ
D.melanogaster_Abd-B  ....TDTSAAVKRHTAHWAYNDEGFNQHYG............................SG
```

```
                      90         100       110                 120
E.sinensis_lab        GMAATQHHVSQSYDYTSAVQAAAVAGGV............GGQTGVMSPA.........G
L.vannamei_lab        GMSAAQHHVTQGYDYTSAVQAAAVAGGV............GGQTGVMSPA..........
D.melanogaster_lab    .........MHDYQMNGQLDMCRGGGGG.............GSGVGNGPV.......GVG
E.sinensis_Dfd        YGRDAMQYNHAGYYQQACVMPQHQPMAA.............HISPQIAPC..........
L.vannamei_Dfd        YGRDAMQYNHAGYYQQTCVMPQHQPMAA.............HMSPQVAPC..........
D.melanogaster_Dfd    HQASAGGYSSNYANATPPSHPHSHPHAHPHQSLGYY....VHHAPEFISA.........G
E.sinensis_Scr        TPSGSGGSSTPGPSVQGRLHQTSSSPAS......TPQAPNSSASCKFAST........PE
L.vannamei_Scr        TPSGSGGNTTPGPSVQGRLHQTSTSPAA.......TPQASSSASCKFAPT.......PES
D.melanogaster_Scr    GVSGSNNNNNSANSNNNNSQSLASPQDL..........STRDISPKLSPS...SVVESVA
E.sinensis_ftz        HSYYPALTSPRGDMTATHPHSAAVPSSA..............VPHAVSPV........NS
L.vannamei_ftz        ATSPLGYDQGHNYYMNSATAARNDEAVA........QAMSQAVPDSLSPV..........
D.melanogaster_ftz    TNPVKKLKYTPDYFYTTVEQVKKAPAVS............TKVTASPAPS....YDQEYV
E.sinensis_Ubx        LYGAPQDHMTPNPFKVDCAKDQNGYGIS............KDMSGGWGPT......VRPA
L.vannamei_Ubx        LYAPPQEHMTPNPFKVDCVKDQNGYGAT............KDMSGGWGPA......VRPA
D.melanogaster_Ubx    GAGGTGGAGNANGGNAANANGQNNPAGG..............MPVRPSAC...TPDSRVG
E.sinensis_Antp       QPHEAHDYRDPSPAAPTCMGQQASPPVQ............QYSSCKMAGQ..........
L.vannamei_Antp       QPHDAHDYRDPSPAAPTCMGQQASPPVQ............QYSSCKMAGQ........QQ
D.melanogaster_Antp   QVTHPQQQQQQPVVYASCKLQAAVGGLG........MVPEGGSPPLVDQMSGHHMNAQMT
E.sinensis_abd-A      SVTSHQLSSTPATNMSPFTAMTANTDGD..............KQCRYSQT........GA
L.vannamei_abd-A      SVTSHQLSANAPSNMSPFSAMTATTDAE..............KQCRYSQT........GA
D.melanogaster_abd-A  RYTDTVMNSYQSMSVPASASAQFAQFYQ.............HATAAASAV.......SAA
E.sinensis_Abd-B      AFAAQTWCNYPPYGRVGHVDAHGQPVPY.............LTAADGAPK...SAVEAAA
L.vannamei_Abd-B      AFAAQTWCNYPPYGRVGHVDAHGQPVPY.............LTAADGAPK...SAMEAAA
D.melanogaster_Abd-B  YYDRKHMFAYPYPETQFPVGQYWGPNYR............PDQTTSAAAA.........A

                      130       140       150          160
E.sinensis_lab        AGGYPGYLDPAAQYRHMSMGLYAHHEGVRD...ACGVGGMMGG.................
L.vannamei_lab        SAGYPGYLDPAAQYRHMSMGLYAHHEGVRE...ACGVGGMMGG.................
D.melanogaster_lab    GNGSPGIGGVLSVQNSLIMANSAAAAGSAH...PNGMGVGLG..................
E.sinensis_Dfd        ...HSPLQQHQVPPRSPVASPDPSAGGTGG...TGSMGAQMAQHMGGGGVEGS.......
L.vannamei_Dfd        ...HSPLQQHQVPPRSPVASPDPSAGG......AGGMGAQMGQHMGGAGVDGS.......
D.melanogaster_Dfd    AVHSDPTNGYGPAANVPNTSNGGGGGGSGAVLGGGAVGGSANGYYGGYGGGYGTANGSVG
E.sinensis_Scr        SSANPVGSPQDLTVTTGGSGPGSSSSGSGSEQNSSSQGGGGSTSNSGGGGEGAEGSESLE
L.vannamei_Scr        STANPVGSPQDLSVSAGGNGPGSSSSGSGSEQNSSSQGGGGGSSNSGGGGEGAEGGESLE
D.melanogaster_Scr    RSLNKGVLGGSLAAAAAAAGLNNNHSGSGV...SGGPGNVNVPMHSPGGGDSDSESDSGN
E.sinensis_ftz        DFNSAKMNAYINPMAGMTPPPSVSAVPSNPLSATDALSHVYGSAAA..............
L.vannamei_ftz        NSDYSSVKMNSYVAANPMAGLTPTPVSSVPAHPLSPSDALSHAYATA.............
D.melanogaster_ftz    TVPTPSASEDVDYLDVYSPQSQTQKLKNGDFATPPPTTPTSLPPLEGISTPPQSPGEKSS
E.sinensis_Ubx        CTPDPVVARGYPPDPSTSPRDRSSHVGAWN...TCGMTPVSQPHQPPQQ...........
L.vannamei_Ubx        CTPDPVAARGYPPDPSTSPRDRAAHVGGWN...TCGMTPVSQPHQQSQ............
D.melanogaster_Ubx    GYLDTSGGSPVSHRGGSAGGNVSVSGGNGN...AGGVQSGVGVAGAGTAWNANCTISGAA
E.sinensis_Antp       GPPQQPQQPQQQQAQQQPPGMDPN..GGPP...QDTSLHMAAQDHQGWPAQQ........
L.vannamei_Antp       GPPQQPQQQPGMDPSGGPPQDTAHHMAAQE.............HGQGWPA..........
D.melanogaster_Antp   LPHHMGHPQAQVHQNHHNMGMYQQQSGVPP...VGAPPQGM..MHQGQGPPQMHQGHPGQ
E.sinensis_abd-A      TDMSQYGLNLQNCATTSNMAQYFHQNNVTNPLNSC.........................
L.vannamei_abd-A      TDMSQYGLNLQNCATTSNMAQYFHQNNATNPLNSCS........................
D.melanogaster_abd-A  SAGAIGVDSLGNACTQPASGVMPGAGGAGG..............................
E.sinensis_Abd-B      GYPHDGYLRNYPTAESMPPAPYPPGCGRAS...AGWLDRAGGSRVSRLPAPRVDAAKDKI
L.vannamei_Abd-B      GYPHDGYLRNYPTAETMPPAPYPPAHR.................................
D.melanogaster_Abd-B  AYMNEAERHVSAAARQSVEGTSTSSYEPPTYSSPGGLRGYPSENYSSSGASGGL......

                        170       180
E.sinensis_lab        ..SPHALPHHQPQQSVP...........................................
L.vannamei_lab        ..SPHGLPHHQPQQTVP...........................................
D.melanogaster_lab    ..SGSGLSSCS.................................................
E.sinensis_Dfd        ..PEETVTELDANGQPV...........................................
L.vannamei_Dfd        ..PEETVTELDANGQPV...........................................
D.melanogaster_Dfd    STHSQGHSPHSQMMDLPLQCSSTEPPTNTALGLQELGLKLEKRIEEAVPAGQQLQELGMR
E.sinensis_Scr        GASGSSGSSSTTKTQPQ...........................................
L.vannamei_Scr        GASGSAGGSSSGKPAPQ...........................................
D.melanogaster_Scr    EAGSSQNSGNGKKNPPQ...........................................
E.sinensis_ftz        ..PDAVMQPYTSKTQCPAN.........................................
L.vannamei_ftz        ..PDAVMQPYTSKTQSPAN.........................................
D.melanogaster_ftz    SAVSQEINHRIVTAPNGAG.........................................
E.sinensis_Ubx        ..QPQQQTNNQMNQPPSNTT........................................
L.vannamei_Ubx        ..QPPQQTNNQMNQAPSNTT........................................
D.melanogaster_Ubx    AQTAAASSLHQASNHT............................................
E.sinensis_Antp       ..PPQQQQNTSSALPSP...........................................
L.vannamei_Antp       ..QQPQQQNTQAALPSP...........................................
D.melanogaster_Antp   HTPPSQNPNSQSSGMPSP..........................................
E.sinensis_abd-A      ..SQPTPTPHIPDIP.............................................
L.vannamei_abd-A      ..QPTAPTPHIPDIP.............................................
D.melanogaster_abd-A  ..........AGIADLP...........................................
E.sinensis_Abd-B      ACKQIYIHPRLPSGVPASG.........................................
L.vannamei_Abd-B      ..PPFPVSHPGALGSNP...........................................
D.melanogaster_Abd-B  ..SVGAVGPCTPNPG.............................................
```

```
E.sinensis_lab          ........................................................TYKW
L.vannamei_lab          ........................................................TYKW
D.melanogaster_lab      ............................................................
E.sinensis_Dfd          ........................................................IYPW
L.vannamei_Dfd          ........................................................IYPW
D.melanogaster_Dfd      LRCDDMGSENDDMSEEDRLMLDRSPDELGSNDNDDDLGDSDSDEDLMAETTDGERIIYPW
E.sinensis_Scr          ........................................................IYPW
L.vannamei_Scr          ........................................................IYPW
D.melanogaster_Scr      ........................................................IYPW
E.sinensis_ftz          ........................................................YYQW
L.vannamei_ftz          ........................................................YYQW
D.melanogaster_ftz      ........................................................DFNW
E.sinensis_Ubx          ........................................................FYPW
L.vannamei_Ubx          ........................................................FYPW
D.melanogaster_Ubx      ........................................................FYPW
E.sinensis_Antp         ........................................................LYPW
L.vannamei_Antp         ........................................................LYPW
D.melanogaster_Antp     ........................................................LYPW
E.sinensis_abd-A        ........................................................RYPW
L.vannamei_abd-A        ........................................................RYPW
D.melanogaster_abd-A    ........................................................RYPW
E.sinensis_Abd-B        ........................................................VYEY
L.vannamei_Abd-B        .........................................................LEW
D.melanogaster_Abd-B    ........................................................LHEW
```

```
                          190       200           210
E.sinensis_lab          MQVKRNVPKPAPKTD............YGGFGGGTGRTNF....................
L.vannamei_lab          MQVKRNVPKPAPKTD............YGGFGGGTGRTNF....................
D.melanogaster_lab      ...........................LSSNTNNSGRTNF....................
E.sinensis_Dfd          MKKIHVAGSEGAGTLYANGQF......QPGCEPKRQRTAY....................
L.vannamei_Dfd          MKKIHVAGAEGAGTLFANGSF......QPGCEPKRQRTAY....................
D.melanogaster_Dfd      MKKIHVAGV.......ANGSY......QPGMEPKRQRTAY....................
E.sinensis_Scr          MKRVHLGQSTV................NSNGETKRQRTSY....................
L.vannamei_Scr          MKRVHLGQNQKDGGKGTV.........NSNGETKRQRTSY....................
D.melanogaster_Scr      MKRVHLGTSTV................NANGETKRQRTSY....................
E.sinensis_ftz          VKAYPAVPNGVGLPAGQ..........DTGAGPKRTRQTY....................
L.vannamei_ftz          VKAYPAAGQ..................EPGSGPKRTRQTY....................
D.melanogaster_ftz      SHIEETLA...................SDCKDSKRTRQTY....................
E.sinensis_Ubx          MAIA.......................GANGLRRRGRQTY....................
L.vannamei_Ubx          MAIA.......................GANGLRRRGRQTY....................
D.melanogaster_Ubx      MAIAGECPEDPTK..............STNGLRRRGRQTY....................
E.sinensis_Antp         MR.........................SQFAERKRGRQTY....................
L.vannamei_Antp         MR.........................SQFAERKRGRQTY....................
D.melanogaster_Antp     MRSQF......................GKCQERKRGRQTY....................
E.sinensis_abd-A        MSITENQWRGLTANWNGLPWS......PNGCPRRRGRQTY....................
L.vannamei_abd-A        MSITENQWRGLTANWNGLPWNFGALRGPNGCPRRRGRQTY....................
D.melanogaster_abd-A    MTLTDWMGSPFERVVCGDFNG......PNGCPRRRGRQTY....................
E.sinensis_Abd-B        LNSEYSLGREPKTCCRDVYSYVFA...LGSLTTCLSPSPYYQPFPLFLFHPSPFLFPSSP
L.vannamei_Abd-B        ...........................TGNMTVRKKRKPY....................
D.melanogaster_Abd-B    ...........................TGQVSVRKKRKPY....................
```

```
                                                220                 230       240
E.sinensis_lab          ....................TTKQLTELEKEFH.........FNKYLTRARRIEIASALQ
L.vannamei_lab          ....................TTKQLTELEKEFH.........FNKYLTRARRIEIASALQ
D.melanogaster_lab      ....................TNKQLTELEKEFH.........FNRYLTRARRIEIANTLQ
E.sinensis_Dfd          ....................TRHQILELEKEFH.........FNRYLTRRRRIEIAHSLC
L.vannamei_Dfd          ....................TRHQILELEKEFH.........FNRYLTRRRRIEIAHSLC
D.melanogaster_Dfd      ....................TRHQILELEKEFH.........YNRYLTRRRRIEIAHTLV
E.sinensis_Scr          ....................TRYQTLELEKEFH.........FNRYLTRRRRIEIAHALC
L.vannamei_Scr          ....................TRYQTLELEKEFH.........FNRYLTRRRRIEIAHALC
D.melanogaster_Scr      ....................TRYQTLELEKEFH.........FNRYLTRRRRIEIAHALC
E.sinensis_ftz          ....................TRYQTLELEKEFH.........FNRYLTRRRRIEIAHALC
L.vannamei_ftz          ....................TRYQTLELEKEFH.........YNRYLTRRRRIEISHALG
D.melanogaster_ftz      ....................TRYQTLELEKEFH.........FNRYITRRRRIDIANALS
E.sinensis_Ubx          ....................TRYQTLELEKEFH.........TNHYLTRRRRIEMAHALC
L.vannamei_Ubx          ....................TRYQTLELEKEFH.........TNHYLTRRRRIEMAHALC
D.melanogaster_Ubx      ....................TRYQTLELEKEFH.........TNHYLTRRRRIEMAHALC
E.sinensis_Antp         ....................TRYQTLELEKEFH.........FNRYLTRRRRIEIAHALC
L.vannamei_Antp         ....................TRYQTLELEKEFH.........FNRYLTRRRRIEIAHALC
D.melanogaster_Antp     ....................TRYQTLELEKEFH.........FNRYLTRRRRIEIAHALC
E.sinensis_abd-A        ....................TRFQTLELEKEFH.........FNHYLTRRRRIEIAHALC
L.vannamei_abd-A        ....................TRFQTLELEKEFH.........FNHYLTRRRRIEIAHALC
D.melanogaster_abd-A    ....................TRFQTLELEKEFH.........FNHYLTRRRRIEIAHALC
E.sinensis_Abd-B        ILPSLHIFPLTLTSGVRFSFTLFSVVYLSPLFLSFSPSSIIPFTSTFTSQKEAPADSSAD
L.vannamei_Abd-B        ....................SKFQTLELEKEFL.........YNAYVSKQKRWELARNLN
D.melanogaster_Abd-B    ....................SKFQTLELEKEFL.........FNAYVSKQKRWELARNLQ
```

```
                          250           260       270                   280       290
                          .             .         .                     .         .
E.sinensis_lab        LNETQ......VKIWFQNRRMKQKKRMKEGLIAPEPPTV......STPNENSNDSDSTAT
L.vannamei_lab        LNETQ......VKIWFQNRRAKDKRLKEAELERQNRPLY.....................
D.melanogaster_lab    LNETQ......VKIWFQNRRMKQKKRVKEGLIPADILTQ.....................
E.sinensis_Dfd        LSERQ......IKIWFQNRRMKWKKDNKLPNTKNVRRKT.............NPAGVTTT
L.vannamei_Dfd        LSERQ......IKIWFQNRRMKWKKDNKLPNTKNVRRKT.....................
D.melanogaster_Dfd    LSERQ......IKIWFQNRRMKWKKDNKLPNTKNVRKKTVDANGNPTPVAKKPTKRAASK
E.sinensis_Scr        LTERQ......IKIWFQNRRMKWKKEHKMASMNAGMGMH.....................
L.vannamei_Scr        LTERQ......IKIWFQNRRMKWKKEHKMASMNAGMGMH.....................
D.melanogaster_Scr    LTERQ......IKIWFQDRRMKWKKEHKMASMNIVPCHM.....................
E.sinensis_ftz        LTERQ......IKIWFQNRRMKAKKESKQSHDSTGEKSE.....................
L.vannamei_ftz        LTERQ......IKIWFQNRRMKAKKESKISSAGGEGGAA.....................
D.melanogaster_ftz    LSERQ......IKIWFQNRRMKSKKDRTLDSSPEHCGAG......YTAMLPPLEATSTAT
E.sinensis_Ubx        LTERQ......IKIWFQNRRMKLKKEIQAIKELNEQEKQ.....................
L.vannamei_Ubx        LTERQ......IKIWFQNRRMKLKKEIQAIKELNEQEKQ.....................
D.melanogaster_Ubx    LTERQ......IKIWFQNRRMKLKKEIQAIKELNEQEKQ.....................
E.sinensis_Antp       LTERQ......IKIWFQNRRMKWKKENKSKV.............................
L.vannamei_Antp       LTERQ......IKIWFQNRRMKWKKENKTKGVEN..........................
D.melanogaster_Antp   LTERQ......IKIWFQNRRMKWKKENKTKGEPGSGGEG.....................
E.sinensis_abd-A      LTERQ......VKIWFQNRRMKLKKELRAVKEINEQVRR...........EREEQDKLKQ
L.vannamei_abd-A      LTERQ......VKIWFQNRRMKLKKELRAVKEINEQVRR.......EREEQEKLKQQQDD
D.melanogaster_abd-A  LTERQ......IKIWFQNRRMKLKKELRAVKEINEQARRDREEQEKMKAQETMKSAQQNK
E.sinensis_Abd-B      SEKRRKNKTTGANFPQQGKKVGAIVVSGRGTKQGERKRADPEPPAVALQSERAGGRGPGQ
L.vannamei_Abd-B      LTERQ......VKIWFQNRRMKNKKNSQRQAAQEAAAAA....................A
D.melanogaster_Abd-B  LTERQ......VKIWFQNRRMKNKKNSQRQANQQNNNNN.....................

                          300       310                                      320
                          .         .                                        .
E.sinensis_lab        TGGSAGGGSAGGSSGGSGGA...........................SGGVTTKPE....
L.vannamei_lab        .......PTYGGLLPMGGLV...........................PSLALQRPL....
D.melanogaster_lab    ....HSTSVISEKPPQQQQPQ..........................PPELQLKSQ....
E.sinensis_Dfd        VTPKNQQQNQNQTQNTTQNP...........................PAQDQQHS.....
L.vannamei_Dfd        ................NPWP...........................PCALK........
D.melanogaster_Dfd    KQQQAQQQQQSQQQQTQQTPVMNECIRSDSLESIGDVSSSLGNPPYIPAAPETTSS....
E.sinensis_Scr        .......PQAYHQMHHQMMH...........................PHHLHPHLA....
L.vannamei_Scr        .......PQAYQHMHHQMMH...........................PHHLHPHLA....
D.melanogaster_Scr    .......GPYGHPCHQFDIH...........................PSQFAHLSA....
E.sinensis_ftz        .......ESEEKEACDISSS...........................PDLLTGALP....
L.vannamei_ftz        GGGGGEESEEKEVSSDPSSS...........................PELLTASLA....
D.melanogaster_ftz    TGAPSVPVPMYHHHQTTAAY...........................PAYSHSHSH....
E.sinensis_Ubx        ........AQNAKLAAQQNA...........................ANNPQAMTP....
L.vannamei_Ubx        ........AQSQKMAQQQQS...........................ASNAQSMTS....
D.melanogaster_Ubx    ........AQAQKAAAAAAA...........................AAAVQ........
E.sinensis_Antp       ........ENGNSLSDTPTP...........................TSPSQ........
L.vannamei_Antp       ..........GNSLSETPTP...........................TSPTQ........
D.melanogaster_Antp   ...............DEITP...........................PNSPQ........
E.sinensis_abd-A      QQDDKKTNKEQQPSTANGQP...........................PSATNGSSS....
L.vannamei_abd-A      KKTNKDQASAGNTPAGAATN...........................ASSSSSSTS....
D.melanogaster_abd-A  QVQQQQQQQQQQQQQQQQQQHQQQQQQPQDHHSIIAHN.........PGHLHHSVVGQND
E.sinensis_Abd-B      RAGSSDMSQKDSKLYNETGSYFIGIN.....................RIGIYDLPK....
L.vannamei_Abd-B      AGTPSSGGGTPGHQPNTPQT...........................PNSIKP.......
D.melanogaster_Abd-B  .....SSSNHNHAQATQQHH...........................SGHHLNLSL....

                                            330       340       350
                                            .         .         .
E.sinensis_lab        ................SPGSGGGGDTQPLQVTPATTKPKTQALPATTQSPS.........
L.vannamei_lab        ................VPLLG.......................................
D.melanogaster_lab    ................GSDLGGNELATGAPSTPTTAMTLTAPTSKQS.............
E.sinensis_Dfd        ............................................................
L.vannamei_Dfd        ............................................................
D.melanogaster_Dfd    ................YPGSQQHLSNNNNNGSGNNNNNNNNNNSNLNNNNNNNQMGHTNL
E.sinensis_Scr        ................DFDTKGYY....................................
L.vannamei_Scr        ................DLETKGYY....................................
D.melanogaster_Scr    ............................................................
E.sinensis_ftz        ................LPLPGPLTVT..................................
L.vannamei_ftz        ................GSLTGTLTGSLTAELTAT..........................
D.melanogaster_ftz    ................GYGLLNDYPQQQTHQQYDAYPQQYQHQCSYQQHPQDLYHLS...
E.sinensis_Ubx        ................NQGAGGGPGADQNSANPAQN........................
L.vannamei_Ubx        ................SQGAGGGAGADQNPPNPTQN........................
D.melanogaster_Ubx    ....................GGHLDQ..................................
E.sinensis_Antp       ............................................................
L.vannamei_Antp       ............................................................
D.melanogaster_Antp   ............................................................
E.sinensis_abd-A      ................SSSAGGGTGDTKAAT.............................
L.vannamei_abd-A      ................SASAGGGAGDTKAAT.............................
D.melanogaster_abd-A  LKLGLGMGVGVGVGGIGPGIGGGLGGNLGMMSALDKSNHDLLKAVSKVNS..........
E.sinensis_Abd-B      ................IEDAGGERGKWKTERLGKMGMEC.....................
L.vannamei_Abd-B      ............................................................
D.melanogaster_Abd-B  ................NMGHHAAKMHQ.................................
```

**E.sinensis_lab**	....
**L.vannamei_lab**	....
**D.melanogaster_lab**	....
**E.sinensis_Dfd**	....
**L.vannamei_Dfd**	....
**D.melanogaster_Dfd**	HGHLQQQQSDLMTNLQLHIKQDYDLTAL
**E.sinensis_Scr**	....
**L.vannamei_Scr**	....
**D.melanogaster_Scr**	....
**E.sinensis_ftz**	....
**L.vannamei_ftz**	....
**D.melanogaster_ftz**	....
**E.sinensis_Ubx**	....
**L.vannamei_Ubx**	....
**D.melanogaster_Ubx**	....
**E.sinensis_Antp**	....
**L.vannamei_Antp**	....
**D.melanogaster_Antp**	....
**E.sinensis_abd-A**	....
**L.vannamei_abd-A**	....
**D.melanogaster_abd-A**	....
**E.sinensis_Abd-B**	....
**L.vannamei_Abd-B**	....
**D.melanogaster_Abd-B**	....
