# Supplementary figures and images for: The identification, adaptive evolutionary analyses and mRNA expression levels of homeobox (hox) genes in the Chinese mitten crab Eriocheir sinensis
Source: BMC Genomics. 2023 Aug 3;24:436. doi: 10.1186/s12864-023-09489-w (PMC10401747; doi:10.1186/s12864-023-09489-w)

Supplementary file S2. Amino acid composition of Hox proteins in *Eriocheir sinensis*

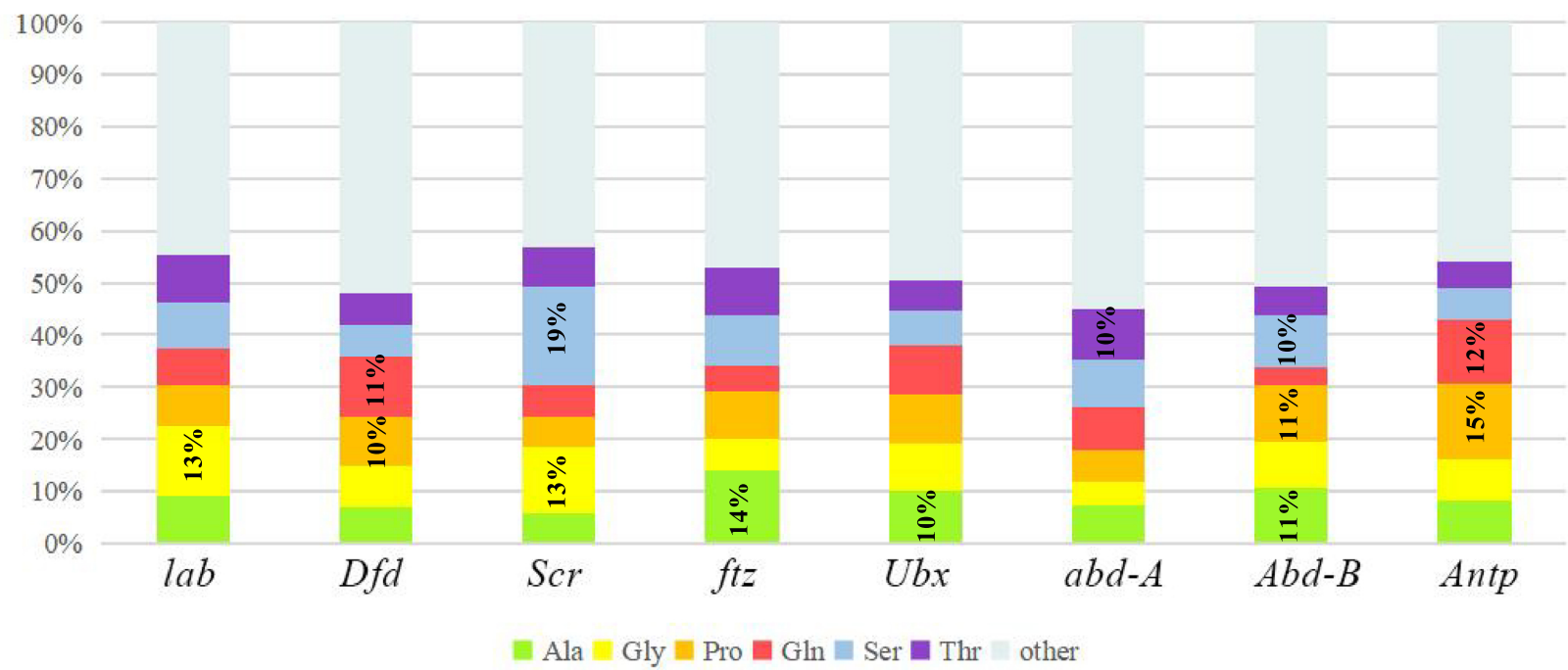

Supplement: Supplementary file 2 — Supplementary Material 2 [file 12864_2023_9489_MOESM2_ESM.pdf]

**SUPPLEMENTARY FILE S5. Domains of Hox genes in 13 species**

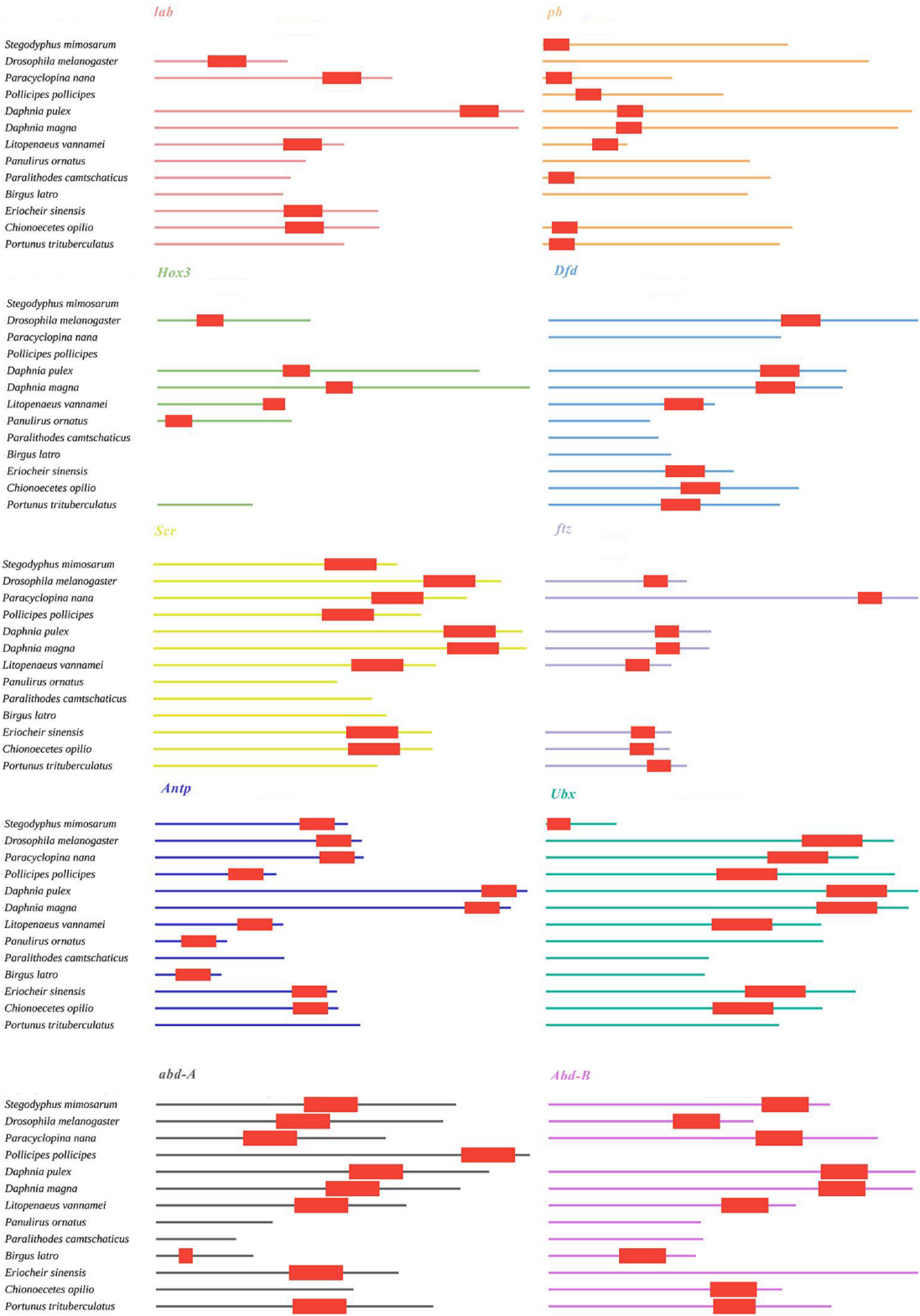

Supplement: Supplementary file 5 — Supplementary Material 5 [file 12864_2023_9489_MOESM5_ESM.pdf]
