# Supplementary material for: The identification, adaptive evolutionary analyses and mRNA expression levels of homeobox (hox) genes in the Chinese mitten crab Eriocheir sinensis
Source: BMC Genomics. 2023 Aug 3;24:436. doi: 10.1186/s12864-023-09489-w (PMC10401747; doi:10.1186/s12864-023-09489-w)

SUPPLEMENTARY FILE S4. The full-length cDNA structure of *Scr* and *Antp* from *Eriocheir sinensis*

a

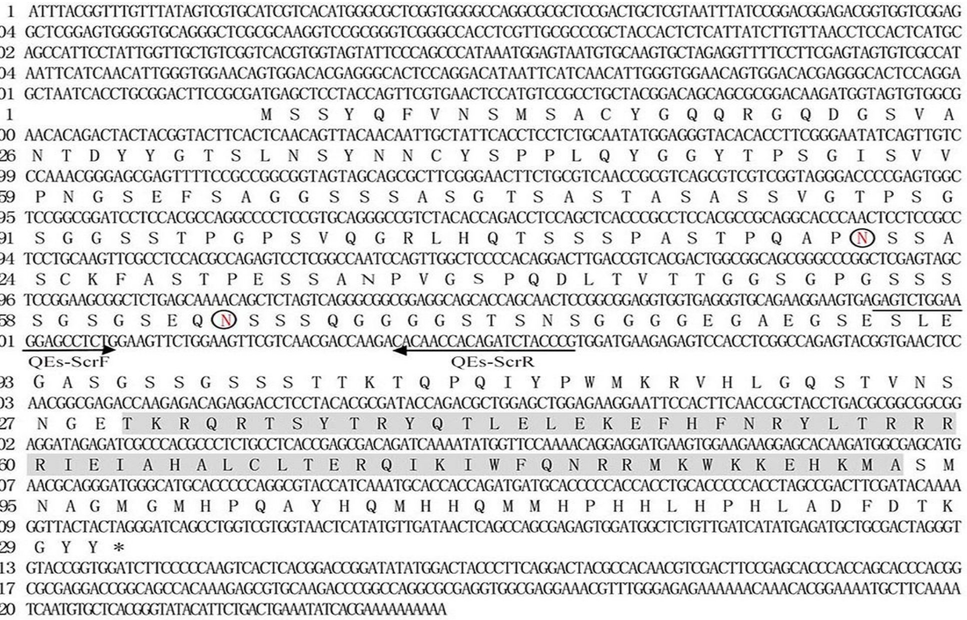

b

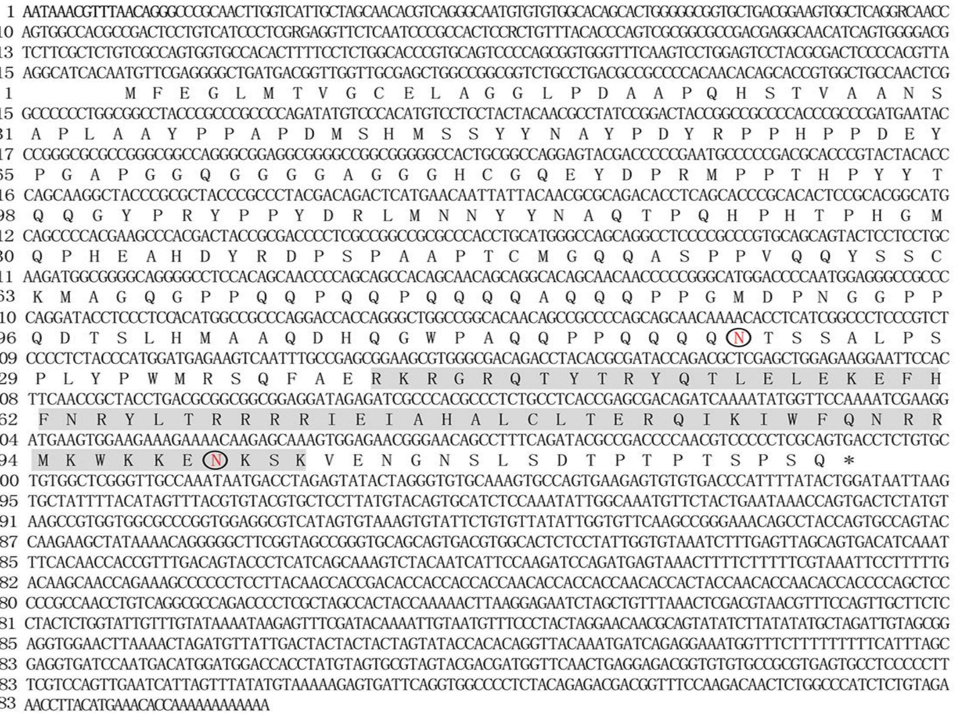

Supplement: Supplementary file 4 — Supplementary Material 4 [file 12864_2023_9489_MOESM4_ESM.pdf]
