# Supplementary material for: The identification, adaptive evolutionary analyses and mRNA expression levels of homeobox (hox) genes in the Chinese mitten crab Eriocheir sinensis
Source: BMC Genomics. 2023 Aug 3;24:436. doi: 10.1186/s12864-023-09489-w (PMC10401747; doi:10.1186/s12864-023-09489-w)
Supplement: Supplementary file 6 — Supplementary Material 6 [file 12864_2023_9489_MOESM6_ESM.docx]

**SUPPLEMENTary FILE S7. Selective pressure analyses of Hox based on branch model (branch Brachyura)**

| Gene | Models  (branch Brachyura) | np | -lnL | LRT P- values | Omega values |
| --- | --- | --- | --- | --- | --- |
| *lab* | one-ratio (M0)  two-ratio | 22  23 | 1455.098  1452.840 | 0.0335 | ω_0_=0.0602  ω_1_=0.1793 |
| *Scr* | one-ratio (M0)  two-ratio | 25  26 | 3344.156  3343.561 | 0.2755 | ω_0_=0.1193  ω_1_=0.1702 |
| *Antp* | one-ratio (M0)  two-ratio | 23  24 | 1464.302  1464.159 | 0.5932 | ω_0_=0.0491  ω_1_=0.0708 |
| *Ubx* | one-ratio (M0)  two-ratio | 17  18 | 2067.615  2061.614 | 0.0005 | ω_0_=0.0228  ω_1_=0.0112 |
| *ftz* | one-ratio (M0)  two-ratio | 15  16 | 4713.532  4712.136 | 0.0948 | ω_0_=0.1003  ω_1_=0.1569 |
| *abd-A* | one-ratio (M0)  two-ratio | 17  18 | 2359.625  2359.402 | 0.5044 | ω_0_=0.0047  ω_1_=0.0022 |
| *Abd-B* | one-ratio (M0)  two-ratio | 20  21 | 2680.479  2680.122 | 0.3983 | ω_0_=0.0225  ω_1_=0.0408 |
