# Supplementary material for: The identification, adaptive evolutionary analyses and mRNA expression levels of homeobox (hox) genes in the Chinese mitten crab Eriocheir sinensis
Source: BMC Genomics. 2023 Aug 3;24:436. doi: 10.1186/s12864-023-09489-w (PMC10401747; doi:10.1186/s12864-023-09489-w)
Supplement: Supplementary file 7 — Supplementary Material 7 [file 12864_2023_9489_MOESM7_ESM.docx]

**Supplement S8 Selective pressure analyses of *Hox* based on branch site model**

| Gene | Models  (branch Brachyura ) | np | -lnL | LRT P- values | positively selected sites (*PP*>0.95) |
| --- | --- | --- | --- | --- | --- |
| *lab* | Model A  Model A null | 25  24 | 1439.082 1441.761 | 0.0206 | 18 A 0.991**,50 T 0.991**,51 N 0.987*,53 T 0.988*,55 K 1.000** |
| *Scr* | Model A  Model A null | 28  27 | 3314.677 3314.771 | 0.6641 | ————  Not allowed |
| *Antp* | Model A  Model A null | 26  25 | 1371.261 1382.933 | 0.0000 | 57 K 1.000** |
| *Ubx* | Model A  Model A null | 20  19 | 2041.543 2041.719 | 0.5534 | 3 S 0.951*,4 Y 0.962*,120 T 0.965* |
| *ftz* | Model A  Model A null | 18  17 | 4639.966 4639.966 | 1.0000 | ————  Not allowed |
| *abd-A* | Model A  Model A null | 20  19 | 2335.214 2338.965 | 0.0062 | 64 G 0.969*,  65 E 0.974*,  67 H 0.981*,  70 S 0.985*,  77 R 0.960*,  78 T 0.960*,  79 K 0.974*,  80 D 0.974*,  82 E 0.980*,  87 S 0.978*,  88 P 0.951*  94 Y 0.973*,  96 R 0.952*,  99 H 0.979*,  100 C 0.972*,  101 T 0.971*,  102 S 0.961*,  104 I 0.956* |
| *Abd-B* | Model A  Model A null | 23  22 | 2678.448 2678.448 | 1.0000 | ————  Not allowed |
